# Liquid Metastable Precursors of Ibuprofen as Aqueous Nucleation Intermediates

**DOI:** 10.1002/anie.201910986

**Published:** 2019-11-06

**Authors:** Eduard Wiedenbeck, Michael Kovermann, Denis Gebauer, Helmut Cölfen

**Affiliations:** ^1^ Physical Chemistry University of Konstanz Universitätsstraße 10 78457 Konstanz Germany; ^2^ Leibniz University of Hannover, Institute of Inorganic Chemistry Callinstraße 9 30167 Hannover Germany

**Keywords:** crystal growth, crystallization, nucleation, organic molecules

## Abstract

The nucleation mechanism of crystals of small organic molecules, postulated based on computer simulations, still lacks experimental evidence. In this study we designed an experimental approach to monitor the early stages of the crystallization of ibuprofen as a model system for small organic molecules. Ibuprofen undergoes liquid–liquid phase separation prior to nucleation. The binodal and spinodal limits of the corresponding liquid–liquid miscibility gap were analyzed and confirmed. An increase in viscosity sustains the kinetic stability of the dense liquid intermediate. Since the distances between ibuprofen molecules within the dense liquid phase are similar to those in the crystal forms, this dense liquid phase is identified as a precursor phase in the nucleation of ibuprofen, in which densification is followed by generation of structural order. This discovery may make it possible to enrich poorly soluble pharmaceuticals beyond classical solubility limitations in aqueous environments.

## Graphical abstract

**The nucleation mechanism** of crystals of small organic molecules was studied for the crystallization of ibuprofen as a model system. Ibuprofen undergoes liquid–liquid phase separation prior to nucleation and forms a dense liquid phase that is identified as a precursor phase in the nucleation of ibuprofen. These results may provide insight for the development of new pharmaceutical formulations.

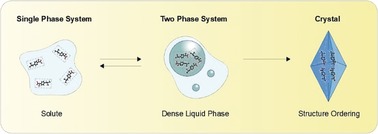

## Introduction

Crystallization, a natural phenomenon observed in everyday life, is crucial to many processes occurring in nature and in the chemical, pharmaceutical, and food industries. The majority of agrochemical and pharmaceutical products undergo many crystallization steps during their development and manufacture, where corresponding processes serve as versatile techniques in separation, purification, and product design.^1^, ^2^ More than 90 % of all active pharmaceutical ingredients (APIs) are small organic molecules in the crystalline state.^2^ Notably, the selection of suitable polymorphs of drug components plays a key role in formulation and manufacturing design, and thus it is crucial for achieving the desired solubility and stability properties.^3^ Since solution crystallization starts with nucleation, early events of nucleation play a decisive role in the generation of the crystal structure and size distribution of generated particles.^4^ Hence, understanding the fundamentals of nucleation and precursor phases to the final crystal is vital to control the properties of the final product.

Nevertheless, the mechanistic understanding of phase separation and the formation of solid particles or liquid intermediates of the final crystalline systems is rather limited, especially for small organic molecules. Due to the analytical simplicity of classical nucleation theory (CNT), researchers have applied it extensively to solution crystallization. CNT considers that clusters of critical size form stochastically in supersaturated solutions due to the reversible addition of single molecules to unstable precritical clusters. Once they reach the critical size, their growth is thermodynamically favorable and as a result of this, crystal growth proceeds up to the final crystal.^5^, ^6^ The general assumption in CNT (capillary assumption) is that the properties of the nucleus can be represented by those of the bulk, for example, macroscopic surface tension.

On the other hand, mechanisms of nonclassical nucleation were explored in simulations by ten Wolde and Frenkel.^7^ Further experiments showed evidence for a two‐step process for protein crystal nucleation in which the separation of a dense, liquid phase is followed by formation of crystalline order inside the liquid precursor.^8^, ^9^, ^10^, ^11^ Computer simulations^12^, ^13^ and experimental studies of protein nucleation in solution^14^, ^15^ suggested that nucleation involves at least two stages. However, recent work also challenged the applicability of the model of two‐step nucleation to the field of protein crystallization.^16^ The prenucleation cluster pathway pioneered by Gebauer et al.^17^ for CaCO_3_ involves the formation of prenucleation clusters in solution, decrease in their dynamics and densification as the key step for phase separation, formation of a liquid phase, solidification, and finally crystallization.^18^ While both two‐step nucleation and the prenucleation cluster pathway consider liquid intermediates, the former mechanism relies on the formation of critical nuclei within the liquid intermediate for crystallization, while the latter is based on the aggregation and dehydration of larger entities that cannot be accounted for in such classical notions of nucleation.^19^, ^20^

However, for small organic molecules the experimental evidence for metastable liquid phases within a two‐step nucleation or a prenucleation cluster pathway is rather poor. Supersaturated glycine solutions underwent laser‐induced nucleation at rates much faster than those of control solutions.^21^ This was explained by the electric‐field‐induced alignment of the molecules in existing prenucleation clusters in solution. The entropic barrier for the formation of an ordered lattice is supposed to be reduced in this way. Nakamura et al.^22^ described the formation of a dense liquid phase mediated by surface interactions with carbon nanohorns. In a second step, heterogeneous nucleation was observed as the precursor phase was attached to the nanostructured surface. Another study reported by Rybtchinski et al.^23^ corroborated a two‐step nucleation pathway for perylene diimides as simple aromatic compounds. Here, the final crystallinity was observed by cryo‐TEM (cryogenic transmission electron microscope) imaging to gradually develop in amorphous precursors rather than at the nucleation stage. Molecular dynamics computer simulations at increasing levels of supersaturation showed that liquid–liquid phase separation occurs before nucleation, independent of the present solvent.^24^ Furthermore, links between the self‐assembly of organic molecules in solution and crystal structure after nucleation were found to depend on the specific solvent used for the nucleation of the organic crystal.^25^, ^26^

The poorly water‐soluble compound ibuprofen is a prominent model system and one of the most frequently prescribed essential analgesics in the world according to WHO.^27^ Commercially it is employed as a racemic crystal while the API is the *S*‐enantiomer.^28^ The final crystal structure formed from aqueous solutions differs depending on whether the racemate or solely the *S*‐enantiomer is employed.^29^, ^30^ Due to the acid functionality of the molecule, the solubility depends strongly on the protonation state, and its supersaturation can be adjusted via the pH value and calculated based on the law of mass action.

Here, we aim to elucidate the underlying nucleation pathway of ibuprofen with emphasis on a liquid precursor phase in aqueous solution. We monitored the early stages of nucleation with a potentiometric titration assay taking advantage of the protolysis equilibrium of ibuprofen in combination with turbidimetry. Furthermore, we characterized the physicochemical properties of this precursor phase such as its molecular dynamics and viscosity. By quantifying the amount of ibuprofen bound within the dense liquid phase via ^1^H NMR spectroscopy, we located the binodal as well as the spinodal limits of the corresponding liquid–liquid miscibility gap at room temperature. Furthermore, we measured the interproton distances in the dense liquid phase by 2D ^1^H‐^1^H NOESY NMR experiments and compared them with interproton distances in the final crystal. This reveals that the liquid intermediates are dynamic species with intermolecular distances similar to those in crystals, where the concentration of the poorly soluble compound is significantly increased.

This inherent property might provide a new means to improve the bioavailability of poorly water‐soluble compounds when liquid intermediates can be stabilized in pharmaceutical formulations.

## Results and Discussion

### Potentiometric Titration Experiments

**Figure 1:**
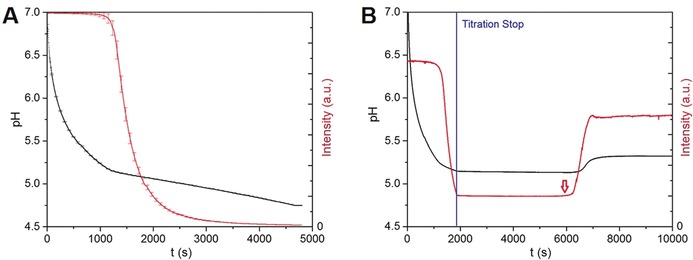
A: Continuous addition of HCl to a solution of *S*‐ibuprofen resulting in decreasing pH (black curve, left scale) and decreasing intensity (intensity decreases with increasing turbidity; red curve, right scale). Error bars represent standard deviations of three independent titrations. B: Exemplary titration with a constant level of turbidity after titration stop (blue line). The red arrow indicates the nucleation point determined from the increase in intensity.

**Scheme 1:**
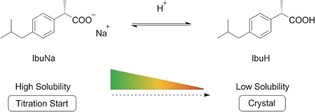
Protolysis equilibrium and change in the solubility of the weak organic acid ibuprofen.

**Scheme 2:**
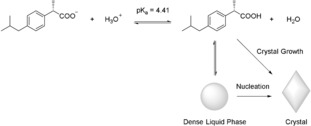
Protolysis equilibrium of ibuprofen with suggested pathway of nucleation and crystal growth.

The supersaturation of protonated ibuprofen (IbuH) was increased by adding dilute hydrochloric acid solution to an ibuprofen sodium solution and decreasing the pH (Scheme 1). Another dosing unit was used to titrate ibuprofen sodium solution in order to maintain a constant overall concentration of ibuprofen throughout the whole experiment. In Figure 1 (left) it can be seen that after a certain point the turbidity increased steadily and reproducibly while HCl was added to the solution. Stopping the addition of HCl (Figure 1, right) after the observed increase in turbidity resulted in a constant level of turbidity (blue line). After a certain stirring time, the pH rose and crystals formed, which were characterized by X‐ray diffraction (Figure S1, Supporting Information). The time point of nucleation could be determined as a slight decrease of turbidity (red arrow), caused by the incorporation of ibuprofen molecules into the forming crystals and—related to this phenomenon—due to the associated withdrawal of protons from solution (Scheme 2). After crystallization and a short period of equilibration, the pH value rose to a constant value which reflects the solubility limit of crystalline ibuprofen in aqueous solution at this pH value.

### Determination of the Locus of the Liquid–Liquid Binodal Limit

**Figure 2:**
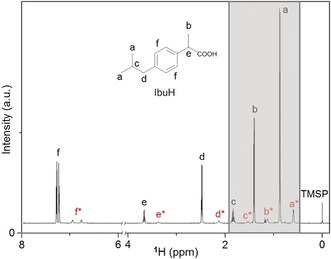
One‐dimensional ^1^H NMR spectrum of a sample removed from a titration experiment at pH 5.0 before nucleation. The peaks were assigned to the ibuprofen molecule, while the additional peaks correspond to ibuprofen in the separated dense liquid phase (labeled with *). The sample was drawn from the titration of *S*‐IbuNa solution (3 mm) with 15 mm HCl solution and 6 mm *S*‐IbuNa solution added at a rate of 0.2 mL min^−1^ using the double‐dosing technique. The spectral range of 0.4–1.9 ppm (grey area) was used for two‐dimensional ^1^H‐^1^H NOESY NMR spectroscopy (Figure 5).

**Figure 3:**
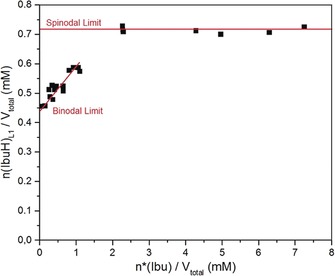
Results from the ^1^H NMR measurements of samples drawn from titration. Here, integrals of proton signals (a) (Figure 2) were determined at different concentrations of ibuprofen in the dense liquid phase. The concentration of ibuprofen *c*(IbuH) in the mother phase after phase separation was calculated from the measured pH value based on Equation (5) (Supporting Information). The liquid–liquid binodal limit was identified as the *y*‐offset from linear regression of the data at low concentrations. The liquid–liquid spinodal limit was identified as a saturation threshold of *n*(IbuH)_L1_/*V*_total_.

For the determination of the locus of the liquid–liquid binodal limit, samples were drawn from titrations performed with the double‐dosing method in 5 vol. % D_2_O and ^1^H NMR spectra were recorded subsequently. While signals of the ibuprofen molecule were present in samples taken before phase separation, additional peaks were present in those samples, in which liquid–liquid phase separation had already occurred. Interestingly, all of the recorded signals of ibuprofen exhibit secondary signals due to the separated phase, shifted by about 0.3 ppm (for aliphatic protons) to about 0.5 ppm (for aromatic protons) (Figure 2). Considering the observed turbidity increase, we suppose that the chemical environment of ibuprofen in the dense phase is different and hence results in the difference in chemical shift. Also, these signals show a lower intensity because of the relatively small amount of ibuprofen present in the dense liquid phase compared to the overall amount of ibuprofen in solution. During the course of titration in the regime of liquid–liquid phase separation, samples were drawn at specific pH values. These samples were investigated in terms of their signal integrals for peaks (a) and (a*) arising from molecules in the mother phase and dense liquid phase, respectively, while their sum always equals the total invariant ibuprofen concentration of 3 mm. The decrease in volume of the mother phase is likely minor and negligible for calculating the concentration of IbuH in this phase (Figure 3). With decreasing pH, the amount of ibuprofen present in the dense liquid phase (n*(Ibu)/*V*_total_) increases with a concurrent decrease of the amount of ibuprofen in the mother phase (*n*(IbuH)_L1_/*V*_total_), whereby the total amount of ibuprofen remains constant; thus, the amount in both phases correlates linearly. Extrapolation of the corresponding linear fit yields the lowest concentration at which liquid–liquid phase separation can occur.

Hence, the *y*‐offset of the linear fit corresponds to the binodal limit of liquid–liquid miscibility gap. On the other hand, a saturation threshold can be identified at much higher supersaturations. This implies that the composition of the dense liquid phase does not change upon further increase in the concentration of IbuH, and thus the saturation threshold corresponds to the locus of the liquid–liquid spinodal limit.

### Translational and Rotational Motion in the Dense Liquid Phase

**Figure 4:**
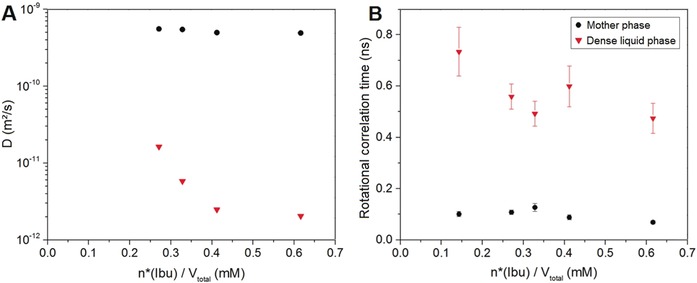
^1^H NMR PFG‐STE self‐diffusion experiment data illustrated in a plot of diffusion coefficients of ibuprofen molecules (A). Rotational correlation time of ibuprofen molecules in both phases (^1^H NMR peaks (a, a*), see Figure 2) plotted vs. the amount of bound ibuprofen in the dense liquid phase (B). Samples were drawn from titration of *S*‐IbuNa solution (3 mm) with 15 mm HCl solution and 6 mm *S*‐IbuNa solution added at a rate of 0.2 mL min^−1^. Error bars represent the standard deviation of the fitting procedure.

In order to study the molecular dynamics of ibuprofen in the dense liquid phase, the pure *S*‐enantiomer of ibuprofen was employed since it exhibits a higher solubility than racemic ibuprofen.^31^ For samples drawn from titration, ^1^H PFG‐STE diffusion NMR was employed as a non‐invasive, highly precise technique for the determination of diffusion coefficients.^32^, ^33^ The diffusion coefficient of ibuprofen molecules in both phases was determined according to Equation (6) (see the Supporting Information) and linear fitting of the corresponding data (Figure S2 in the Supporting Information). This procedure yielded the same diffusion coefficient *D* for ibuprofen in the mother phase for several different samples drawn in the binodal regime (Figure 4, left). However, the diffusion coefficient of the ibuprofen molecules in the dense liquid phase (labeled as D*) decreases with an increase in the amount of ibuprofen in the dense liquid phase. While the ratio of diffusion coefficients (D/D*) is 34 upon crossing the binodal limit, it increases to a ratio of 239, advancing further into the binodal regime (Table 1). This shows that with an increasing amount of ibuprofen bound in the dense liquid phase, the translational diffusion of molecules in this phase becomes significantly slower. Moreover, NMR provides evidence for the liquid character of the separated phase. Further information on molecular dynamics can be obtained from the rotational correlation time of the ibuprofen molecules in the distinct phases by determining the *T*_1_ and *T*_2_ relaxation times associated with the peaks (a) and (a*) in the one‐dimensional ^1^H NMR spectrum (Figure S2, Supporting information). According to Carper et al.^34^ [Eq. (9), Supporting Information), this yields the rotational correlation times *τ*_c_ (Figure 4, right), which strongly increase by a factor of 5–7 in the dense liquid phase, whereas *τ*_c_ does not change significantly in the mother phase.

**Table 1:** Diffusion coefficients of ibuprofen molecules obtained from the ^1^H NMR PFG‐STE self‐diffusion experiments. Errors refer to standard deviations from fitting procedure.

n*(IbuH)/*V* [mm]	*D* [m^2^ s^−1^]	D* [m^2^ s^−1^]	*D*/D*
0.271	555±2×10^−12^	16.3±0.2×10^−12^	34
0.328	546±3×10^−12^	5.8±0.1×10^−12^	94
0.412	498±1×10^−12^	2.48±0.04×10^−12^	201
0.616	491±2×10^−12^	2.05±0.02×10^−12^	239

The overall results show that the molecular dynamics, both in terms of translation and rotation, are slowed down in the dense liquid phase. While D/D* increases by a factor of 34–239, rotational correlation times *τ*_c_ increase only by a factor of 5–7 in the dense liquid phase.

Consequently, rotational and translational motions of the molecules in the dense liquid phase do not sense the same viscosity since both parameters scale linearly with viscosity according to the Stokes–Einstein and Stokes–Debye equation, respectively. In other words, translational and rotational diffusion cannot be correlated with each other in this system. Similar observations were made by Pielak et al.^35^ for solutions of proteins in the presence of solubilized polymers. We assume that the microviscosity experienced by ibuprofen molecules in the dense liquid phase, as assessed by ^1^H NMR spectroscopy, differs from the bulk viscosity, and thus does not strictly obey Stokes laws. Nevertheless, it is clear that the viscosity in the dense liquid phase is much higher than in the mother phase (D/D* 34–239), which may explain why the liquid phase is kinetically stabilized for a significant period of time before it crystallizes. Viscosity was proposed to play a key role in the formation of the ordered nucleus within a dense liquid intermediate by Vekilov et al.^8^

### Determination of Intermolecular Distances

In order to gain quantitative information about inter‐ vs. intramolecular distances of ibuprofen molecules in the dense liquid phase, two‐dimensional homonuclear ^1^H‐^1^H NMR NOESY methodology (nuclear Overhauser effect spectroscopy) was employed. Solely the protons (a), (b), (c) are relevant here, because they cannot come closer due to the rigid aromatic ring structure and hence, they have a intermolecular distance higher than 5 Å in any conformation. Owing to the general limitations of NOESY methodology, the maximum separation that leads to cross peaks is about 5 Å.^36^

**Figure 5:**
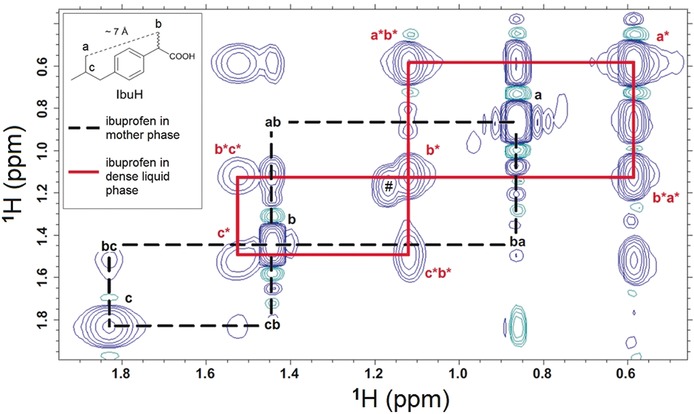
Two‐dimensional ^1^H‐^1^H NOESY NMR spectrum of *S*‐ibuprofen solution after liquid–liquid phase separation (*c*_tot_=3 mm, pH 2.31) in the binodal regime. Red letters indicate the assignment to the proton signals according to Figure 2, whereas the asterisk (*) labels the corresponding proton signal in the dense liquid phase. Intermolecular NOE signals that are present in the dense liquid phase are indicated by red lines, whereas missing intermolecular NOE signals in the mother phase are indicated by black dotted lines. Signals present in the indirect dimension close to the diagonal signals of (a), (a*), and (b) are due to *t*_1_ noise. The signal labeled with a hash (#) originates from trace EtOH.

In samples drawn after liquid–liquid phase separation, no NOE signal can be detected between the protons of the α‐methyl group (a) and those of the isopropyl group (b, c) for the ibuprofen molecules present in the mother phase (Figure 5, inset, dotted line mode). The distance from (a) to (b) in the molecule itself is at their closest distance ≈7 Å. This implies that they are too far away from each other to contribute to a NOE cross signal, and as expected, no NOE signal of (a) to (b) could be observed in the ^1^H NMR NOESY spectrum (Figure 5).

Interestingly, there are NOE signals observed for the proton pairs (a*)–(b*) and (a*)–(c*) in the dense liquid phase, although the intramolecular distance remains about 7 Å. Hence, this NOE signal necessarily arises from an intermolecular NOE with protons of other neighboring ibuprofen molecules closer than 5 Å. In order to obtain a more precise value for the intermolecular proton distance, the NOE signals can be used to determine interproton distances with high accuracy.^37^ As the NOE signal intensity scales with distance, *d* ^−6^, the intermolecular proton distances of the ibuprofen molecules in the same phase can be determined according to [Eq. (1)]:

(1) $$d_{H-H}=\sqrt[{6}]{\frac{I_{\mathrm{ref}}}{I_{H-H}}d_{\mathrm{ref}}}$$

where *d*_H‐H_ is the intermolecular distance of two protons, *I*_H‐H_ is the corresponding signal intensity in the 2D ^1^H‐^1^H NOESY spectrum, *d*_ref_ is the intramolecular distance in the reference molecule, and *I*_ref_ is the corresponding signal intensity in the ^1^H NOESY spectrum. In the case of ibuprofen in the dense liquid phase, the fixed distance between protons (a) and (c) (2.456 Å) within the ibuprofen molecule is chosen as an intramolecular reference (*d*_ref_) with the signal intensity of the (a*)–(c*) NOE signal (*I*_ref_). The results of this NOE interproton distance calculation are summarized in Table 2.

**Table 2:** Results from the NOE distance calculations for distances *d*_a*‐b*_ and *d*_b*‐c*_ calculated with Equation (1).

n*(Ibu)/*V*_total_ [mm]	*d*_a*‐b*_ [Å]	*d*_b*‐c*_ [Å]
0.163	2.04	2.71
0.222	2.04	2.65
0.40	1.97	2.71
0.501	1.97	2.74
2.277	1.97	2.79

These as‐determined distances can be contrasted with those of the protons in the crystal structures of both *S*‐IbuH and the racemic *RS*‐IbuH (Table 3). The intermolecular distances between protons (a) and (b), and (b) and (c) in the crystal forms of IbuH are about 2.44–2.58 Å for (a)–(b) and 3.17–4.98 Å for (b)–(c).^29^, ^30^ Since these values correspond to the intermolecular proton–proton distances in the dense liquid phase, it can be concluded that the ibuprofen molecules in the dense liquid phase are roughly as close to each other as in the ibuprofen crystal.

**Table 3:** Intramolecular and intermolecular distances of (a)–(b) and (b)–(c) protons in the crystal structures of *S*‐IbuH^30^ and racemic IbuH (*rac* IbuH).^29^

	(a)–(b) *rac* IbuH	(b)–(c) *rac* IbuH	(a)–(b) *S*‐IbuH	(b)–(c) *S*‐IbuH
*d* [Å] (intramolecular)	5.50	6.61	5.49	6.21
*d* [Å] (intermolecular)	2.44	4.98	2.58	3.17

However, it has to be mentioned that within the (a*)–(c*) NOE signal there are also contributions of the (a*)–(c*) intermolecular NOE signals. But as the NOE signal intensity scales with *d* ^−6^, their contributions are supposed to be minimal and thus can be neglected. Besides, the NOE signals originating from the close distance to other ibuprofen molecules are the sum of different interproton distances. As their individual NOE signal intensities again scale with *d* ^−6^, closer distances are the major contributors to the total NOE signal. Thus, the calculated values represent fixed interproton distances for ibuprofen when the molecules are organized in clusters with discrete interproton distances. Since it is not known whether this situation is representative for the real scenario, the data provides at least a qualitative measure of an increased aggregation of ibuprofen molecules in the dense liquid phase when compared to the mother phase.

## Conclusion

In summary, nucleation of ibuprofen in aqueous solution proceeds via liquid–liquid phase separation as a precursor to crystals. The loci of the binodal (0.43±0.01 mm) and spinodal limits (0.71±0.01 mm) of the corresponding liquid–liquid miscibility gap were determined by means of ^1^H NMR spectroscopy due to the different chemical environment of the ibuprofen molecules in the two phases. The evaluation of molecular dynamics suggests that both translational and rotational diffusion of ibuprofen in the dense liquid phase are strongly hindered compared to the ibuprofen molecules in the mother phase. This shows that the ibuprofen molecules in the dense liquid phase, which is more concentrated than the mother phase, are in a state that is kinetically stabilized. Since rotational and translational diffusion processes are slowed down significantly, it affects the crystalline structure generation within the dense liquid phase and accounts for its metastability towards nucleation. The intermolecular distances in the dense liquid phase were found to be similar to those in the final crystal according to the measured interproton NOE signals. Consequently, the dense liquid phase can be regarded as a densified precursor phase for the nucleation of a crystalline phase. In the case of ibuprofen, these results show that densification is followed by structure generation and therefore implies a nonclassical nucleation pathway where the dense liquid phase is kinetically stabilized by the slow rotational and translational diffusion.

To the best of our knowledge this is the first time that molecular dynamics and intermolecular distances in a metastable precursor phase have been characterized directly by NMR spectroscopy. The application of the methods presented in this work to other compounds will further contribute to a better understanding of the nucleation process of small organic molecules from solution. Moreover, the existence of this miscibility gap can be exploited in order to reach drug concentrations far above the solubility limit of the crystalline compounds. Therefore, these results have the potential to pave the way towards the fabrication of liquid‐phase drug formulations with an enhanced bioavailability.

## Conflict of interest

The authors declare no conflict of interest.

## Supporting information

As a service to our authors and readers, this journal provides supporting information supplied by the authors. Such materials are peer reviewed and may be re‐organized for online delivery, but are not copy‐edited or typeset. Technical support issues arising from supporting information (other than missing files) should be addressed to the authors.

Supplementary
