# Supplementary material for: Liquid Metastable Precursors of Ibuprofen as Aqueous Nucleation Intermediates
Source: Angew Chem Int Ed Engl. 2019 Nov 6;58(52):19103–9. doi: 10.1002/anie.201910986 (PMC6972611; doi:10.1002/anie.201910986)
Supplement: Supplementary file 1 — Supplementary [file ANIE-58-19103-s001.pdf]

Angewandte Chemie
Eine Zeitschrift der Gesellschaft Deutscher Chemiker

Supporting Information

Liquid Metastable Precursors of Ibuprofen as Aqueous Nucleation Intermediates

Eduard Wiedenbeck, Michael Kovermann, Denis Gebauer,* and Helmut Cölfen**

anie_201910986_sm_miscellaneous_information.pdf

SUPPORTING INFORMATION

Table of Contents

Experimental Section 2
- Materials 2
- Potentiometric titration experiments 2
- The double-dosing method 2
- ^{1}H NMR experiments 2
- Calculation of ibuprofen species concentration 3

Results and Discussion 3
- Titration of ibuprofen: XRD characterization of the ibuprofen precipitates 3
- ^{1}H NMR spectroscopy: PFG-STE self-diffusion NMR 4
- Relaxation times T_1 and T_2 and rotational correlation time τ_c 4

Author Contributions 5

Experimental Section

Materials

The following chemicals were purchased and used as received. (S)-(+)-Ibuprofen (>98.0%) was purchased from TCI Europe. 3-(Trimethylsilyl)-1-propionic-2,2,3,3-d4 acid sodium salt (TMSP, >98.0%) was purchased from Sigma Aldrich. Sodium hydroxide standard solution (0.1 M) and hydrochloric acid standard solution (0.1 M) were purchased from Merck. MilliQ water was used in all experiments. All glassware was cleaned by rinsing with ethanol and water followed by drying before use.

Potentiometric titration experiments

Titration experiments were performed in a computer-controlled titration system supplied by Metrohm (Filderstadt, Germany), with corresponding commercial software (Tiamo™, current version: 2.3). The setup consisted of a titration instrument (Titrando 905, Metrohm No. 2.905.0020) that controlled two dosing devices (Dosino 800, Metrohm No. 2.800.0010), which operated two 807 dosing units with 2 ml volume each (Metrohm No. 6.3032.120). Titrations were performed in a titration vessel with thermostat jacket (Metrohm No. 6.1418.250). The whole assembly was run by an independent power supply, which was necessary to decouple the electric circuit of the instruments from that of the laboratory to avoid artifacts caused by voltage fluctuations. A special double junction pH electrode with 3 M KCl solution as reference electrolyte was employed (EtOH-trode, Metrohm No. 6.0269.100). In order to measure the turbidity during titration, an appropriate optical sensor (Optrode, Metrohm No. 6.1115.000) was used at an emission wavelength of 640 nm. The vessels were closed with a titration vessel lid (Metrohm No. 6.1414.010) and largely decoupled from the atmosphere to minimize potential pH changes that may arise from in-diffusion of atmospheric CO_2 or evaporation of the solutions during the titration experiment. Calibration of the pH electrode was performed at 25.0 ± 0.1 °C.

The double-dosing method

80 ml of 3 mM ibuprofen sodium salt solution at pH 6.8 ± 0.1 were filled into the beaker. In order to induce supersaturation of poorly water-soluble protonated ibuprofen (**Scheme 1**), 15 mM HCl solution was added. At the same time, 6 mM ibuprofen sodium salt solution was added so as to counter-balance the dilution of ibuprofen during HCl addition. Both solutions were dosed at a steady dosing rate of 0.2 ml/min if not stated otherwise. In that way, a constant concentration of the overall ibuprofen species in the analyte solution was achieved. The titration experiments for the preparation of specimens for NMR spectroscopy were carried out at a constant concentration of 5 vol% D_2O.

^{1}H NMR experiments

All NMR experiments were conducted on a Bruker Avance III 600 MHz system using a CP-TCI probe. Samples of 600 µl were drawn from titration experiments and filled into 5 mm (outer diameter) NMR tubes. All one-dimensional ^{1}H NMR spectra were recorded at a temperature of T = 295 K. Deuterium oxide was used as field-lock at a volume fraction of 5 % of the total sample, being held constant throughout all titrations by applying the double-dosing method as described above. Deuterated TMSP was used in all experiments as reference for the proton dimension. Raw data was processed using TOPSPIN software and data fitting was performed in ORIGIN software applying a Levenberg-Marquardt algorithm.

Integrals for proton signals I were determined in the spectral range between 0.551 - 0.634 ppm for ibuprofen in the mother phase and 0.839 - 0.901 ppm for ibuprofen molecules in the dense liquid phase, respectively. These methyl group signals (labeled as a, a^* in the mother phase and dense liquid phase, respectively, possessing six protons per molecule) were analyzed for the determination of the liquid-liquid binodal and spinodal limits as well as for relaxation time measurements and diffusion experiments because of the largest signal-to-noise ratio compared to other signals.

The longitudinal relaxation time, T_1, was determined by using the inversion recovery experiment. Relaxation delays of 30, 100, 300, 1000, 3000 and 10000 ms were used.

The transversal relaxation time, T_2. of ibuprofen in solution as well as in the dense liquid phase was determined by using a Carr–Purcell–Meiboom–Gill (CPMG) sequence. Relaxation delays of 30, 100, 300, 1000, 3000 and 10000 ms were used. The delay between successive π-pulses was set to 1 ms.

Pulsed field gradient stimulated echo experiments (PFG-STE) were used to determine the translational diffusion coefficient D. Hence the diffusion time Δ was set to Δ = 200 ms and the gradient pulse time δ for dephasing and rephasing along the z-axis was set to δ = 3 ms. The gradient field pulse G, was varied between 1 % and 95 % to generate 22 proton spectra slices for further analysis. Gradients were calibrated as described elsewhere.[38]

Two-dimensional ^{1}H-^{1}H NOESY NMR spectra of ibuprofen were recorded by using a mixing time of 1000 ms, 16 scans per increment (1024 points in the direct dimension, 256 points in the indirect dimension, spectral width of 7812 Hz used in both dimensions). Intermolecular distances between two protons d_i were calculated using a value of d representing intramolecular isopropyl protons at a fixed distance of 2.456 Å.[29] Consequently, this fixed distance enables the calculation of d_i of intermolecularly interacting ibuprofen protons since the NOE signal is proportional to d^{-6} (assuming the same rate of molecular tumbling of the two molecules contributing to the build-up of the intermolecular NOE).

Calculation of ibuprofen species concentration

By applying the double-dosing method, the overall concentration of ibuprofen is kept at a constant level and can be calculated during the course of titration according to:

$$c_{total} = [IbuH] + [Ibu^{-}] \quad (2)$$

where c_{total} is the overall concentration of ibuprofen in solution, and [IbuH] and $[Ibu^-]$ correspond to the total concentrations of protonated and deprotonated ibuprofen in the same volume, respectively.

As the pH changes during the course of titrations, the relationship between the pH value and the fraction of the ibuprofen concentrations is determined via the K_a value ($pK_a = 4.41$) and the equation:

$$[H^{+}] = K_a \left(\frac{[IbuH]}{[Ibu^{-}]}\right) \quad (3)$$

By substituting $[Ibu^-]$ using equation (2) and rearrangement the concentration of the protonated ibuprofen in the single-phase system can be calculated as follows:

$$[IbuH] = \frac{c_{total}\frac{[H^{+}]}{K_a}}{1 + \frac{[H^{+}]}{K_a}} \quad (4)$$

For ^{1}H NMR spectroscopy, two phases are present, so the amount of ibuprofen in the dense liquid phase has to be subtracted, since it is not present in the mother phase anymore:

$$\frac{n(IbuH)_{L1}}{V_{total}} = \frac{\frac{[H]^{+}}{K_a}\left(c_{total} - \frac{n^{*}(Ibu)}{V_{total}}\right)}{1 + \frac{[H]^{+}}{K_a}} \quad (5)$$

where $n^{*}(Ibu)$ corresponds to the amount of ibuprofen in the dense liquid phase, determined by ^{1}H NMR spectroscopy in the total volume V_{total} of the solution, while $n(IbuH)_{L1}$ corresponds to the amount of protonated ibuprofen in the mother phase.

Results and Discussion

Titration of ibuprofen: XRD characterization of the ibuprofen precipitates

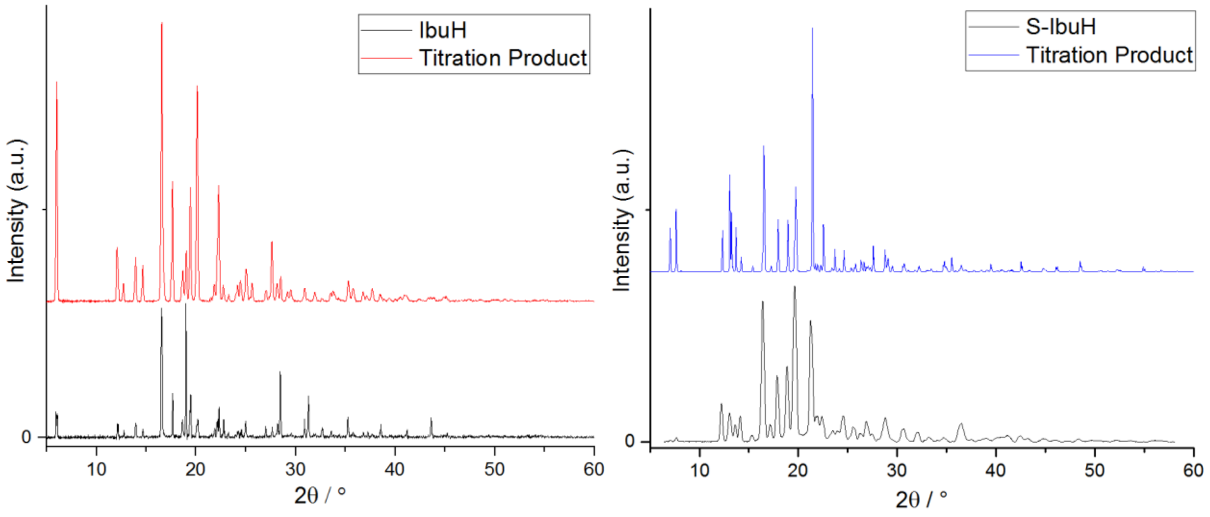

Figure S1. X-ray diffraction graphs of ibuprofen precipitates from titration. Precipitate from titration when racemic ibuprofen is employed (left, red) and precipitate from titration when solely S-Ibuprofen is employed (right, blue) compared with underlaid diffraction peaks of the commercially available IbuH (black, left) and S-IbuH (black, right), respectively.

^{1}H NMR spectroscopy: PFG-STE self-diffusion NMR

Translational motion of the molecule during the diffusion time (*d20*) results in an attenuation of the intensity of signal (A/A_0), which scales linearly with *D* according to the following equation:

$$ln\left(\frac{A}{A_0}\right) = -D(\delta\gamma G)^2\left(\Delta - \frac{\delta}{3}\right) \quad (6)$$

where *D* is the diffusion coefficient, *G* is the gradient field strength, *γ* is the gyromagnetic ratio of ^{1}H, ***δ*** is the duration of the applied gradient application (*p30*) and *Δ* is the diffusion time (*d20*). Plotting these data on a logarithmic scale of signal intensity yields a linear relationship (**Figure S1**).

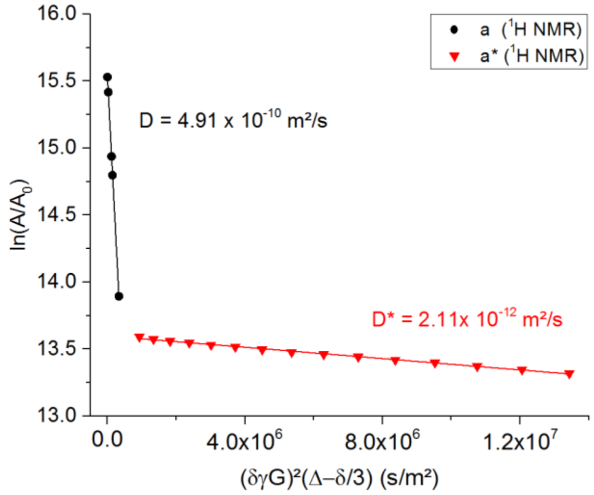

Figure S2. The results of the ^{1}H PFG-STE self-diffusion experiment with the diffusion measurement plot of the degree of attenuation vs. $(\delta\gamma G)^2(\Delta-\delta/3)$, where the slope of the linear fit is - D. For each sample in the binodal regime of ibuprofen two different diffusion coefficients were found, depending on the magnitude of the gradient strength.

Relaxation times T_1 and T_2 and rotational correlation time τ_c

This methodology measures the reduction in peak intensity (M_0) in dependency on the changing delay time τ and the longitudinal relaxation time T_1 and the transversal relaxation time T_2 of the ibuprofen molecule in the respective phase:

$$M_z = M_0\left(1 - 2 \cdot exp\left(-\frac{\tau}{T_1}\right)\right) \quad (7)$$

$$M_{xy} = M_0 \cdot exp\left(-\frac{\tau}{T_2}\right) \quad (8)$$

By fitting an exponential function to the data, T_1 and T_2 relaxation times of ibuprofen in the respective phase can be obtained. The results show that the average T_1 and T_2 relaxation is decreased significantly in the dense liquid phase (**Figure S2**). This indicates that the ibuprofen molecules in the emergent phase are tumbling at a different rate than those in the mother phase. To explore the molecular dynamics of ibuprofen further, the specific rotational correlation times (τ_c) were calculated by inserting T_1 and T_2 relaxation times in a polynomial according to Carper et al.[34]

$$\tau_c(ns) = -0.12044 + 0.197251\frac{T_1}{T_2} - 0.0153\left(\frac{T_1}{T_2}\right)^2 + 0.000666\left(\frac{T_1}{T_2}\right)^3 - 0.000011\left(\frac{T_1}{T_2}\right)^4 \quad (9)$$

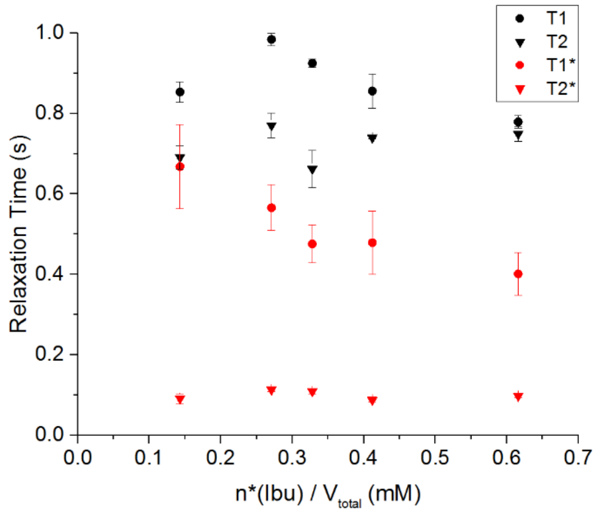

Figure S3. The results of the T_1 and T_2 relaxation time measurements (black = mother phase, red = dense liquid phase) obtained by fitting an exponential function to signal intensities of protons *a, a** at different delay times τ. The relaxation times are plotted vs. the amount of bound ibuprofen in the dense liquid phase, which was determined by ^{1}H NMR.

Author Contributions

H.C. developed the basic project idea and was in charge of the project administration. E.W., H.C., D.G. and M.K. wrote the manuscript (equal). E.W. and H.C. acquired the funding. E.W. conducted the experiments and analyzed the data. H.C., D.G. and M.K. discussed the data (equal).
